# Characterizing highly conserved fragments in 3′UTRs via statistical and transfer learning approaches

**DOI:** 10.1093/nargab/lqag086

**Published:** 2026-07-30

**Authors:** Eric S Ho, Ash Baeck-Hubloux, Nathan Dinh, Ava Severino, Ciara Troy

**Affiliations:** Department of Biology, Lafayette College, Easton, PA 18042, United States; Department of Biology, Lafayette College, Easton, PA 18042, United States; Department of Computer Science, Lafayette College, Easton, PA 18042, United States; Department of Biology, Lafayette College, Easton, PA 18042, United States; Department of Biology, Lafayette College, Easton, PA 18042, United States

## Abstract

3′ Untranslated regions (3′UTRs) serve as regulatory platforms that modulate steps in the central dogma through the binding of RNA-binding proteins and miRNAs. Their binding sites are often identified through orthologous regions among species. A separate but related discovery was the ultraconserved elements (UCEs) detected in human, rat, and mouse genomes two decades ago. However, knowledge about their functions is limited. Perplexingly, mutagenesis of UCEs produced no observable phenotypic differences. The majority of UCEs are non-coding, though ∼8% are located in the 3′UTRs. Given the importance of 3′UTRs in gene regulation, we use a computational approach to identify highly conserved fragments (CFs) that exhibit ≥50 bp and ≥90% identity in 3′UTRs across diverse mammals. CFs are neither composed of simple repeats nor low-complexity regions common to mammalian genomes. Using a transformer-based model, CFs are characterized as A/T-rich and distinguishable from the 3′UTR background. CFs of 100 genes possess conserved RNA structures or are depleted of variation or both. Intriguingly, these genes are enriched in neuronal tissues and play roles in neurodevelopment and RNA processing. Our findings expand on existing studies that attribute enhancer function to UCEs, suggesting a new avenue for exploring the biological roles of CFs in 3′UTRs.

## Graphical Abstract

**Graphical Abstract:**
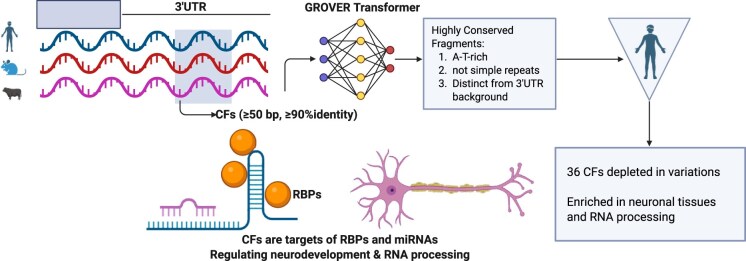

## Introduction

3′ Untranslated regions (3′UTRs) harbor diverse regulatory elements that modulate essential biological functions, including mRNA stability, mRNA localization, and translation (reviewed in [1]). These functions are delivered through sequence-specific binding of RNA-binding proteins (RBPs) to these regulatory elements, followed by the recruitment of effectors by the bound RBPs. Notably, different combinations of RBPs and effectors can produce diametrically opposite effects. For example, AU-rich-binding proteins TTP and KHSRP can mediate exosome complex binding, leading to mRNA degradation [2]. On the other hand, the AU-rich-binding protein HuR (or ELAVL1) is unable to bind the exosome complex, thereby sequestering HuR-bound mRNA from degradation [3]. Post-transcriptionally, 3′UTRs contain sequence elements targeted by miRNAs [4, 5], thereby attenuating translation and mRNA stability [6, 7].

Moreover, alternative polyadenylation is prevalent in human and mouse genes, ∼54% and 32%, respectively [8], creating an additional mechanism to regulate genes through the shuffling in and out of regulatory elements in the long and short 3′UTR isoforms, even if the coding sequence (CDS) remains intact [9]. For example, CD47 localization is regulated by alternative polyadenylation. The CD47 proteins expressed from long 3′UTRs (∼3.5 kb) are anchored on the cell membrane, while CD47 proteins from the short 3′UTR isoform (∼300 bp) are localized mainly in the endoplasmic reticulum [10].

It has been established that non-coding variants are associated with diseases [11–13]. Examples include high-throughput assays developed to screen for variants in 3′UTRs [14] and a cancer mutation burden metric focused exclusively on variants within UTRs [15]. These efforts underscore the critical regulatory roles of 3′UTRs, implying that investigating the conservation of their sequences may offer insights into their functions. Unlike protein-coding regions (CDSs), 3′UTRs are largely unconserved. Regulatory RNA elements in the 3′UTRs are usually short (<10 bp). For instance, the motif of class I AU-rich element is AUUUA, and class II is UUAUUUA(U/A)(U/A). However, in this study, we have discovered long (>50 bp) conserved fragments (CFs; >90% identity) in the 3′UTRs among diverse mammals (human, mouse, rat, cow, and platypus), leading to the question of whether their conservation underpins crucial fundamental functions prevailing in mammals, or even vertebrates.

Previous studies have reported the presence of DNA ultraconserved elements (UCEs) in mammals and vertebrates [16, 17]. Close to 500 UCEs were first reported in humans, mice, and rats, all of which are identical in length (>200 bp). With the availability of more genomes, subsequent studies have found over 14 000 UCEs >100 bp in at least three of the five mammals (human, mouse, rat, cow, and dog). However, only ∼8% of UCEs are reported in the 3′UTR versus ∼28% in introns of protein-coding genes, and ∼17% in 5′UTRs [18, 19].

UCEs are found predominantly in non-coding regions, including introns, splice sites of protein-coding genes, and lncRNA exons (reviewed in [20]). Some UCEs are even found in the intergenic areas, though it is unclear whether they are transcribed [19]. Their peculiar conservation has prompted speculation about their vital functions. However, the current picture remains perplexing. Enhancer is one of the functions being studied experimentally [21, 22], although perfect sequence identity is not required for transcription factor binding. A study found that alternation of ultraconserved enhancers produced no overt phenotypes [23]. A recent study has reported that somatic mutations of certain non-coding UCEs are common in cancers [24]. Genes possessing UCEs are frequently implicated in skeletal muscle and neurological development [22, 25].

Given the essential role of 3′UTRs in gene regulation and the puzzling view of UCEs, both the author [26] and others [27] have reported the presence of highly conserved elements in the 3′UTRs in vertebrates. With the advances of deep learning, we aim to offer a renewed characterization of the CFs present in 3′UTRs, beyond simple categorization of UCEs. By taking a contrastive approach, this study has revealed fundamental differences between CFs and their flanking unconserved 3′UTR sequences. Such understanding might reveal obscure functional elements for RBPs and miRNAs. Although the CFs reported here show >90% identity, rather than 100% as reported in previous UCE studies, we argue that this threshold aligns with the nature of 3′UTR regulatory elements in which sequence variability is permissible for factor binding, such as RBPs and miRNAs. Such a view is also supported by previous studies [25, 28–30]. To ensure that our operating threshold does not lead to excessive false positives, we implemented stringent filtering criteria (detailed below).

With the advanced foundation models in genomics, we used the GROVER model [31] to build a binary classifier to distinguish CFs from 3′UTR sequences harboring no CFs, namely non-CFs. Importantly, by interpreting the model’s internals, i.e., the feature weights, we uncovered subtle sequence properties pertaining to CFs, shedding light on the unique propensities of CFs. In this article, we examine CFs from four perspectives: first, nucleotide propensities, complexity, and distinctive sequence elements. Second, we examine conserved RNA secondary structures detected in CFs. Third, we focus on human CFs that are depleted of variations in the human population. Lastly, we explore the shared tissues, processes, and functions of genes harboring such CFs. Results suggest that the nucleotide composition of CFs differs from that of 3′UTRs, that genes harboring CFs are enriched in neuronal tissues, and that their functions are associated with neurodevelopment and transcription. Our findings guide further investigation and characterize functional roles of CFs in 3′UTRs.

## Materials and methods

### Untranslated regions and coding sequences

A schematic diagram of the processes developed in this study is shown in Supplementary Fig. S1.

We selected five diverse mammalian species to form the core group for analysis: *Homo sapiens* (human, 9606), *Mus musculus* (mouse, 10 090), *Rattus norvegicus* (rat, 10 116), *Bos taurus* (cow, 9913), and *Ornithorhynchus anatinus* (platypus, 9258). The common names and taxonomy IDs are inside the parentheses. Their UTRs were downloaded from UTRdb [32] in Nov 2024. A custom program was developed to select the longest 3′UTR isoform for each gene, resulting in 97 455 (human), 57 187 (mouse), 28 889 (rat), 16 636 (cow), and 12 503 (platypus) 3′UTR sequences.

For the sequence complexity analysis (described below), human CDS and gene information were downloaded from the UCSC Genome Browser [33] in Nov 2024. A customized program was used to tag each 3′UTR sequence with the official gene symbol.

### Homology information

Gene homology information was obtained from the NCBI Gene ftp site: https://ftp.ncbi.nlm.nih.gov/gene/DATA/. Two files, gene_info.gz and gene_orthologs.gz, were downloaded in May 2025. Human genes were used as the lead to identify homologous genes in the other four targeted species. A custom program was developed to select, merge, and organize gene homology information, producing 15 460 homologous gene clusters in which 2902, 7349, and 5209 clusters consisted of three, four, and five species, respectively.

### 3′UTR alignment and conserved fragment identification

MAFFT v7.525 (2024/Mar/13) [34] was used to align the 3′UTRs in each homologous gene cluster with at least three sequences (species) using default parameters, except that the maximum iteration was set to 1000 (“--maxiterate 1000″).

A window-based approach was used to detect CFs in the alignments by examining the proportion of perfect matches within 20-bp windows. In the first step, it marked all positions with perfect matches, i.e., positions where the same nucleotide occurred in all aligned sequences. In the second step, it calculated the proportion of identical matches within a 20-bp window. All positions within the window were marked as conserved if the proportion was ≥0.9. This step was repeated by moving the window along the sequence, one base at a time, from 5′ to 3′ until the end of the alignment. After that, overlapping, marked windows were merged into longer fragments, namely CFs. Using this method, human CFs 50 bp or longer were detected in 2905 gene clusters, constituting the CF50 group. The longest CF was 624 bp. We also used two other cutoff lengths: 100 and 200 bp, to create the CF100 and CF200 groups. The CF100 group contains 760 CFs from 514 genes, whereas the CF200 group contains 123 CFs from 105 genes. See Supplementary Table S1 for the list of genes harboring CFs.

Simulated 3′UTRs were created by randomly shuffling the actual 3′UTRs within each homologous gene cluster. The simulated sequences were grouped into 2902, 7349, and 5209 clusters, consisting of three, four, and five species, respectively, matching the numbers of the real gene clusters. The exact procedure used to identify CFs for real 3′UTRs was applied to simulated 3′UTRs.

## Blastn

To expand the search for CFs beyond the five targeted mammals and non-human primates, we used human CFs with a minimum length of 100 bp (*n* = 760) to query the NCBI RefSeq database [35] using BLASTN 2.17.0 [36]. These are the non-default parameters used in the search: database: RefSeq RNA; excluded all five targeted mammals above and primates; excluded predicted sequences (XP/XM); excluded environmental samples; optimized for highly similar sequences; and turned off low-complexity. BLAST hits with a minimum coverage of 90% were retained for analysis. In total, 165 CFs were found beyond the five targeted mammals. *Sus scrofa* [37], *Equus caballus* [38], and *Canis lupus familiaris* [28] showed up the most. See Supplementary Table S2.

### Positional distribution of CFs in 3′UTRs

Only human and mouse CFs were explored. The genomic locations of CFs from the CF50 group and 3′UTRs were mapped to the human genome (hg38) and the mouse genome (mm39) using the standalone BLAT v. 39x1 [38]. The outputs were kept in BED format. As the lengths of 3′UTRs vary, all 3′UTRs were standardized to a length of 100. As such, the locations of CFs were rescaled between 1 and 100, 1 being the leftmost position right after the stop codon, and 100 being the rightmost position, i.e., the 3′-end of a 3′UTR.

### Dinucleotide composition analysis

To reveal CF’s sequence properties, we compared CF’s dinucleotide composition with human 5′UTRs, CDS, and 3′UTRs. Human CDSs were downloaded from UCSC Genome Browser in Nov 2024. In total, 75 671 CDSs were used in this study. A customized Python program was developed to break down sequences into dinucleotides.

The R pairwise Wilcoxon Rank-Sum test [pairwise.wilcox.test()] was used to test the difference in medians of the four sequence types (3′UTR, 5′UTR, CDS, and CF) for each dinucleotide. The *P*-values were adjusted for multiple testing by the Benjamin–Hochberg method.

The magnitude of differences was supported by effect sizes. The effect size was calculated by the wilcox_effsize() function provided by the R package rstatix, where the reference group was CF. In other words, the effect sizes were calculated individually for each comparison between CF and 3′UTR/5′UTR/CDS.

### t-SNE plot

t-SNE plot was produced using the R package Rtsne [39]. Sequences were transformed into vectors of *k*-mer proportions. For instance, for *k* = 2, a sequence is represented as a 16-dimensional vector. In this study, we created a t-SNE plot for four types of human sequences: CF50 group, CDS, 5′UTR, and 3′UTR. The number of CFs included for CF50, CF100, and CF200 was 2905, 760, and 123, respectively. Because the number of other sequence types was far greater than the number of CFs, 1000 CDS, 5′UTR, and 3′UTR sequences were randomly selected for analysis.

The maximum number of iterations was set to 1500 such that the error converged to a stable value. We tried different perplexity values (5 to 50 in steps of 5) and settled on 20 for the current t-SNE plot.

### Benchmark sequences

Additionally, we synthesized low-complexity sequences consisting of 1 (SIM1NT), 2 (SIM2NT), 3 (SIM3NT), and 4 (SIM4NT) nucleotides. In each case, nucleotides in each position are independent of their neighboring nucleotides, and the sequence length was 1000 bp.

Simulated 3′UTR sequences were generated according to the observed nucleotide composition of the human 3′UTRs, where A, C, G, and T occur 27.5%, 22.5%, 23.5%, and 26.5%, respectively. Each simulated 3′UTR was 1000 bp long. They were grouped to mimic real gene clusters, producing 5363, 7354, and 2808 clusters consisting of five, four, and three species, respectively. In total, 15 525 fictitious clusters were created, the same number as the real gene clusters.

### Complexity of DNA sequences

Byte pair encoding (BPE) was used to assess the complexity of human CDSs, 5′UTRs, 3′UTRs, and simulated sequences. Briefly, the input sequences contain only four letters: “A,” “C,” “G,” and “T.” BPE starts by substituting the most frequent nucleotide pair (dinucleotide) in the input sequences with an unused letter. For example, if “TT” occurs most often, then all “TT”s are substituted with an unused letter, say “B.” Such substitution is recorded and reported when the process that necessitates decompression finishes. The substitution step will repeat until either the unused letters are exhausted or a preset number of rounds of substitution has been reached (the option we chose). The compression ratio is defined as

$1 - \frac{{\mathrm{compressed}\ \mathrm{length}}}{{\mathrm{original}\ \mathrm{length}}}$ . BPE was applied to SIM1NT, SIM2NT, SIM3NT, SIM4NT, CF50, CF100, CF200, CDSs (*n* = 19 439), 5′UTRs (*n* = 15 187), and 3′UTRs (*n* = 18 949). The minimum length of sequences was 100 bp. Note that the start and stop codons of CDSs were removed before BPE processing.

### RepeatMasker

We used RepeatMasker (Galaxy Version 4.1.5+galaxy0) [40] on the Galaxy platform [41] to examine low-complexity regions and repeats using the DFam database [42] for the human species. Two-thousand nine-hundred five human CFs from the CF50 group (259 504 bp) were scanned. For comparison purposes, 2000 samples of CDSs (3 579 255 bp) and 2000 samples of 3′UTRs (10 463 bp) were randomly selected from human sequences. See Supplementary Table S4.

## Grover

The GROVE pre-trained model was installed from https://huggingface.co/PoetschLab/GROVER. Our model was developed on a GPU server with GPU Tesla T4, and CUDA version 12.7. These additional Python libraries were installed:

captum==0.8.0

peft==0.17.1

scikit-learn==1.7.2

scipy==1.15.3

torch==2.6.0+cu124

torchaudio==2.6.0+cu124

torchmetrics==1.8.2

torchvision==0.21.0+cu124

transformers==4.57.1

Positive samples were taken from the CF50 group, which contains 2989 human CFs. For the negative samples, 3′UTRs confirmed with no CFs were randomly selected, resulting in 5203 sequences. Importantly, the lengths of the negative sequences were maintained the same as those of the positive samples. For example, if the length of a positive sequence is 213 bp, we splice a 213-bp 3′UTR segment from a random 3′UTR, starting at a random position. As recommended by GROVER, 100 bp were padded at the beginning and end of both positive and negative sequences. It was done by mapping the genomic coordinates of CFs to hg38 using standalone BLAT v. 39x1, and the output .BED file was uploaded to the UCSC Genome Browser [43] as a custom track. Then, used the menu options Tools/Table Browser/Choose the newly created custom track/Download sequence to add 100 bp before and after the sequence.

Ninety percent and 10% of samples were used for training and validation, and holdout testing, respectively. During training, the positive and negative datasets were balanced by randomly selecting 2989 sequences from the negative training set. A five-fold cross-validation was used to construct the model. Below are the hyperparameters used:

train_args = TrainingArguments(

output_dir=“.”,

per_device_train_batch_size = 64,

eval_strategy=“epoch”,

learning_rate = 5e-5,

num_train_epochs = 10,

lr_scheduler_type=“cosine”,

warmup_ratio = 0.1,

)

LoRA (Low-Rank Adaptation) was used for fine-tuning the model in a low-GPU power environment [44]. Below are the parameters:

lora_config = LoraConfig(

r = 32,

lora_alpha = 64,

target_modules=[“query”, “key”, “value”, “dense”],

task_type = TaskType.SEQ_CLS,

)

The model’s accuracy was evaluated using F1, Matthews Correlation Coefficient (MCC), Precision, and Recall. They are defined below:


\begin{eqnarray*}
\mathrm{Precision} = \ \frac{{\mathrm{ TP}}}{{\mathrm{ TP} + \mathrm{ FP}}},
\end{eqnarray*}

\begin{eqnarray*}
\mathrm{Recall} = \ \frac{{\mathrm{ TP}}}{{\mathrm{ TP} + \mathrm{ FN}}},
\end{eqnarray*}

\begin{eqnarray*}
\mathrm{ F1} = \frac{{2 \times \mathrm{Precision} \times \mathrm{Recall}}}{{\mathrm{Precision} + \mathrm{Recall}}},
\end{eqnarray*}

\begin{eqnarray*}
\mathrm{ MCC} = \ \frac{{\mathrm{ TP} \times \mathrm{ TN} + \mathrm{ FP} \times \mathrm{ FN}}}{{\left( {\mathrm{ TP} + \mathrm{ FP}} \right)\left( {\mathrm{ FP} + \mathrm{ FN}} \right)\left( {\mathrm{ FN} + \mathrm{ FP}} \right)\left( {\mathrm{ FN} + \mathrm{ FN}} \right)}},
\end{eqnarray*}

where TP is true positive, FP is false positive, TN is true negative, and FN is false negative.

The metrics’ statistics were collected by repeating the training step 10 times.

### Integrated gradients

Explainability analysis was performed using Integrated Gradients [45]. Since we trained 10 models above, the average attribution for each token (*n* = 605) was calculated for each model. We then selected the top 10 tokens with the highest median attribution across the 10 models. See Supplementary Table S5.

### Identification of top tokens’ neighbors

A custom Python program was developed for this task. First, it loaded a trained/fine-tuned CF classification model and the tokenizer. Next, it retrieved the BERT embedding via the model. And then, it processed the top 15 tokens identified by Integrated Gradients mentioned above one by one. For each token, it used the torch.topk() function to determine the *k* closest neighbors, where *k* = 5. As such, 64 tokens were identified in total: 15 are the top tokens, and 49 are their neighbors. Note that some top tokens are also neighbors of other top tokens. See Supplementary Table S6.

### Finding tokens in training samples

Human CF50 sequences (*n* = 2989) and segments of human 3′UTRs with no CFs (*n* = 5203) were selected if they achieved a prediction score ≥0.8. A custom Python program was developed to tokenize these sequences using the same tokenizer associated with the trained model. The 64 tokens were tallied across the tokenized sequences. Refer to Supplementary Table S7 for the counts and proportions.

### Proportion test

The R prop.test() function was used to test whether the proportion of the 64 tokens is the same between the CFs and non-CFs samples used to train the GROVER model. Recall that the number of CFs and non-CFs samples is 2989 and 5203, respectively. The counts, proportions, *P*-values, and −log10 *P*-values are provided in Supplementary Table S7.

### CFs intolerant of variations in humans

Two distinct constraint metrics were used in this study: JARVIS [41] and UK Biobank depletion rank score (DR) [46]. Both metrics were downloaded from UCSC Genome Browser for the human genome (hg38) in Dec 2025. They were converted from bigWig to bedgraph using the UCSC GB tool bigWigToBedgraph. Next, they were mapped with the genomic coordinates of CFs from the CF50 group, and the median score was calculated for each CF. A custom Python program was developed to select intolerant CFs with a JARVIS score of at least 0.9998 and a DR of no more than 0.1, yielding 36 intolerant CFs across 25 genes. They are listed in Supplementary Table S9.

### Mapping RNA-binding proteins binding sites

RBP binding results from eCLIP experiments were downloaded from ENCODE [47] in July 2025. In total, there are 250 RBP datasets, of which 145 are from K562 and 105 are from HepG2. The downloaded binding results were already mapped to the human genome hg38. To identify RBPs that bind to CFs, we mapped CFs to the human genome hg38 using standalone BLAT v. 39x1. And then, bedtools intersect [48] was used to determine genomic coordinates overlapping between RBPs and intolerant CFs. The -F option was 1.0 to ensure that the entire RBP binding site falls within the CF. See Supplementary Table S9 for details.

### Mapping human miRNAs

Genome (hg19) locations of human predicted (conserved) targets of conserved miRNA families were downloaded from https://www.targetscan.org/cgi-bin/targetscan/data_download.vert80.cgi in Dec 2025. The UCSC Genome Browser LiftOver tool was used to translate genomic coordinates from hg19 to hg38. bedtools intersect was used to determine genomic coordinates that overlap between miRNAs and intolerant CFs. The -F option was set to 1.0 to ensure that the entire miRNA-binding site falls within the CF. See Supplementary Table S9 for details.

### RNA secondary structures of CFs

ViennaRNA-2.7.0 RNAfold [49] was used to predict the structure of two groups of human transcripts: full-length transcripts and 3′UTRs. The former was downloaded from NCBI: https://ftp.ncbi.nlm.nih.gov/refseq/MANE/MANE_human/release_1.4/MANE.GRCh38.v1.4.refseq_rna.fna.gz. Default parameters were used for the predictions.

A one-dimensional convolutional neural network (1D-CNN) was developed using Pytorch to differentiate secondary structure exhibited in CFs and non-CFs. The positive and negative training datasets were adopted from GROVER. The original sequences and predicted structures of CFs and non-CFs, along with a 100-bp flanking region, were extracted from RNAfold outputs for model training. The 1D-CNN model comprised two convolutional layers (input channels = 7, filter size = 5, number of filters = 128, hidden dimensions = 64, and dropout rate = 0.5), followed by a fully connected linear layer with ReLU as the activation function. The seven channels represented the four nucleotides, and the folding structure symbols “.”, “(“, and “)”. The model was trained on the GPU server as GROVER. A five-fold cross-validation was used to train the model, with a batch size of 32, 40 epochs, and a learning rate of 5e-5. The same assessment metrics used by GROVER were calculated on 10 repeated training trials.

### Conserved RNA structures

In addition to the 1D-CNN, conserved RNA structures were mapped to mouse CFs using the predicted conserved structure data (*n* = 26 188) from Seemann *et al*. [50]. Additional file 1 from the article was downloaded from the publisher’s website in June 2026. The genomic coordinates of structures found in 3′UTRs (*n* = 22 808) were converted from the mouse genome mm8 to mm39 using the LiftOver tool by UCSC Genome Browser [43]. They were then intersected with the mouse CFs (*n* = 3118) from the CF50 group using bedtools intersect, with the fraction of overlap (-F) set to 1.0 [48]. See Supplementary Table S8.

### Functional enrichment analysis

R Enrichr package [51] was used for enrichment analysis. Enrichr analysis requires setting up background genes. We chose human protein-coding genes as the background genes. All human gene symbols were downloaded from https://ftp.ncbi.nlm.nih.gov/gene/DATA/GENE_INFO/Mammalia/Homo_sapiens.gene_info.gz in Sep 2025. After filtering out non-coding genes, ribosomal RNAs, and genes that have not yet been assigned an official gene symbol (LOC), the background gene set contained 19 473 human genes. The general process involved selecting databases based on the category of interest. In our case, we focused on tissue enrichment, GO Process enrichment, and GO Molecular Function enrichment. The database choices are listed in the main text. In all categories, only terms with adjusted *P*-value <.05 were considered.

Odds ratios for enrichment were calculated based on Fisher’s exact test as follows:

**Table:** 

	In tissue/GO	Not in tissue/GO
In gene set	*a*	*b*
Not in gene set	*c*	*d*


\begin{eqnarray*}
\mathrm{Odds}\ \mathrm{Ratio}\ \left( {\mathrm{ OR}} \right) = \ \frac{{a \times d}}{{b \times c}}\ ,{{\ \mathrm{ where}\ N}} = {{a\ }} + {{\ b\ }} + {{\ c\ }} + {{\ d}}.
\end{eqnarray*}

For large sample sizes (*N*), the natural log (ln) of the OR is approximately normally distributed. Thus, the standard error (SE) can be determined as follows:


\begin{eqnarray*}
\mathrm{ SE}\left( {\ln \left( {\mathrm{ OR}} \right)} \right) = \ \sqrt {\frac{1}{a} + \frac{1}{b} + \frac{1}{c} + \frac{1}{d}}.
\end{eqnarray*}

The confidence interval at significance level α is


\begin{eqnarray*}
{\mathrm{exp}}\left[ {\ln \left( {\mathrm{ OR}} \right) \pm {{Z}_{1 - {\raise0.7ex\hbox{$\alpha $} \!\mathord{\left/ {\vphantom {\alpha 2}}\right.} \!\lower0.7ex\hbox{$2$}}}} \times \mathrm{ SE}\left( {\ln (\mathrm{ OR})} \right)} \right].
\end{eqnarray*}

### Last exon expression of intolerant genes

Recall that the longest isoform of a gene was used to identify the CFs. CFs were always found in the last (3′-most) exon. We manually checked that CFs were located in the last exon of intolerant genes on the GTEx Portal (https://gtexportal.org/home/) (last accessed in April 2026).

To obtain the expression of the last exon in 54 non-disease tissues, we used the R package gtexr (version 0.2.1) [52], with the default dataset version (datasetId) gtex_v8. The get_gene_search() function was used to retrieve the Ensembl gene IDs (with versions) for the 25 intolerant genes. get_median_exon_expression() function was used to retrieve the median expression (unit in readcount) of each Ensembl gene ID from non-disease tissues. Expressions of the last exon in non-disease tissues were extracted for each intolerant gene, and they can be found in Supplementary Table S12. The number of brain or nerve tissues among the top 10 most highly expressed tissues was tallied and is shown in Table 8.

### GeneAgent

We used GeneAgent web tool [53] to elucidate the concerted function of miRNAs or RBPs that bind to intolerant CFs. GeneAgent uses a Large Language Model (LLM) for gene set analysis (GSA); distinct from statistics-based GSA tools, such as GSEA [54], DAVID [55], etc. The GeneAgent website (https://www.ncbi.nlm.nih.gov/CBBresearch/Lu/Demo/GeneAgent/geneagent.html) was assessed in January 2026. In each submission, we included the gene harboring an intolerant CF and the miRNAs or RBPs that bind to it. GeneAgent processed the information in five steps: Initial generation of the gene set, self-verification of the process name, modification, self-verification of the analytical narratives, and summarization. In total, 30 sets of genes and miRNAs/RBPs were submitted.

## Results

### Long conserved fragments in 3′UTRs are unusual

**Figure 1.**
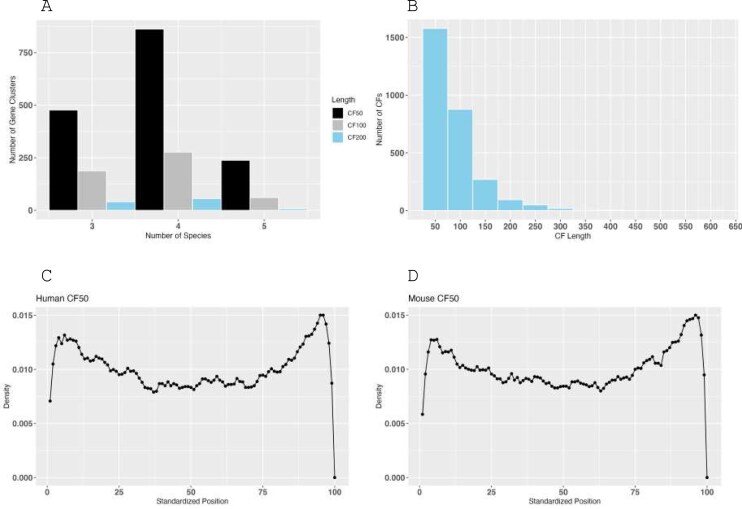
Basic statistics of CFs. (**A**) Distribution of gene clusters containing CFs. The bars represent the number of gene clusters—categorized by species count (three, four, or five)—that contain at least one CF of length ≥50 bp (black), ≥100 bp (gray), or ≥200 bp (blue). (**B**) Distribution of CF lengths. (**C**) Positions where human CFs (≥50 bp) are detected with the longest 3′UTR as the reference. The length of a 3′UTR is standardized to 100, where positions 1 and 100 are the 5′-end and 3′-end of a 3′UTR, respectively. (**D**) Same as panel (C) for the mouse.

We selected a core set of species to identify long CFs based on the availability of information and diversity: human, mouse, rat, cow, and platypus. Human 3′UTRs and homologous 3′UTRs from at least two of the non-human targeted species, i.e. mouse, rat, cow, and platypus, were grouped into clusters per gene, resulting in 15 460 gene clusters, where gene clusters are categorized by the number of constituent species (three, four, or five), depending on the available homology information. Specifically, there are 2902, 7349, and 5209 three-species, four-species, and five-species gene clusters, respectively. 3′UTRs in each gene cluster were aligned by MAFFT [34], followed by percentage of identity and length filtering. Aligned fragments that exhibit 90% or more identity and a minimum length of 50 bp, 100 bp, or 200 bp were extracted into three CF groups, namely CF50, CF100, and CF200. Note that CF50 is the biggest group as it includes CFs from CF100 and CF200. Similarly, CF100 is a superset of CF200. The distribution of gene clusters containing CFs is shown in Fig. 1A. Approximately 17% (477 of 2902), 12% (862 of 7349), and 5% (238 of 5209) of gene clusters contain at least one CF. The majority of the gene clusters consist of four core species. The length distribution of the CF50 group is shown in Fig. 1B.

Table 1 shows the number of genes and CFs for the three CF groups in humans. The complete list of gene symbols for the three groups is provided in Supplementary Table S1. The longest CF is 620 bp and comes from PURA (purine-rich element binding protein A), and shares 97% identity among human, mouse, rat, and cow. In terms of the total length of CFs per gene, RC3H2 (ring finger and CCCH-type domains 2) has the most extended total length of 2739 bp, originating from 12 CFs found in the gene (Supplementary Table S1).

**Table 1.** Human CFs statistics

	CF50	CF100	CF200
Number of genes	1430	514	105
Number of CFs	2905	760	123
Median length (bp)	72	133	244

To eliminate the concern that the observed CFs are spurious, we applied the exact procedure for identifying CFs on simulated 3′UTR sequences in which nucleotides of real 3′UTRs were randomly shuffled, ensuring their length and nucleotide composition were maintained. No fragments with length ≥50 bp that showed ≥90% identity were found, ascertaining that the occurrence of CFs by chance is statistically improbable.

### Prevalence of CFs beyond the targeted mammals

We are wondering whether CFs are present beyond the five targeted mammals. Hence, 760 human CFs (≥100 bp) were used to query cDNAs deeper into other non-primate mammals deposited in the NCBI RefSeq RNA database [35] using BLASTN [56]. Hits that covered at least 90% of the query CF were retained for analysis. In total, 165 out of 760 human CFs were identified in other mammals, where 60 CFs were found in *Sus scrofa*, 37 in *Equus caballus*, and 28 in *Canis lupus familiaris*. Intriguingly, the CFs of two antizymes, AZIN1 (antizyme inhibitor 1) and OAZ2 (ornithine decarboxylase antizyme 2), were found in 47 and 36 mammals, respectively (see Supplementary Table S2). When the search was expanded to non-mammalian species, we found CFs even in chicken (*Gallus gallus*) and the African clawed frog (*Xenopus laevis*). This indicates that CFs are more widespread in the 3′UTRs.

Remarkably, AZIN1 and OAZ2 work in concert to regulate ornithine decarboxylase (ODC), a vital enzyme in polyamine biosynthesis. Polyamines (putrescine, spermidine, and spermine) are small, cationic organic molecules present in all cell types. They play a role in cell growth and proliferation; dysregulation of polyamines can lead to tumor growth [57, 58]. ODC activity is inhibited by OAZ2 upon binding, thereby promoting its degradation. In turn, OAZ2 is negatively regulated by AZIN1, indirectly activating ODC. These three enzymes form an autoregulation mechanism for maintaining polyamine levels in cells.

As CFs of AZIN1 and OAZ2 are widespread in mammals, we found that they are associated with RBPs and miRNAs, thereby altering mRNA stability and translation. RBPs from ENCODE eCLIP [47] and miRNAs from TargetScan [59] were mapped to the CFs. AZIN1 carries nine CFs with a median size of 139 bp (Supplementary Table S1). Seventy RBPs were mapped to AZIN1 (Supplementary Table S11), including HNRNPK, IGF2BP1, PUM1, PUM2, TIA1, TIAL1, and FUS, which bind AU-rich elements in neuronal cells [37]. Twelve miRNAs were mapped to AZIN1’s CFs. Notably, a cancer-linked miR-433-3p was mapped, which might interfere with polyamine regulation.

Since OAZ2 contains only one CF of size 112 bp, only 12 RBPs were mapped to the CF. They are involved in mRNA turnover (KHSRP, PABPC4, and LARP4), translation control (DDX3X, DHX30, and IGF2BP1), and neuronal processes (QKI, DDX3X, DHX30) [60–63]. Two miRNAs were mapped to OAZ2’s CF: miR-365-3p and miR-452-5p/892-3p. At present, their biological functions remain unclear.

CFs of two key regulators of polyamine homeostasis, AZIN1 and OAZ2, exhibit deep evolutionary conservation across highly divergent mammalian lineages. These observations exemplify the regulatory potential of CFs. As experimentally validated sequence data (not from computer prediction) from non-model organisms become increasingly available, comparative analyses of these fragments may reveal evolutionarily preserved post-transcriptional regulatory architectures and provide mechanistic insight into the selective forces responsible for their remarkable conservation.

### CFs concentrate at 3′UTRs’ ends

CFs are found mainly localized at the two ends of the 3′UTRs, as shown in Fig. 1C and D. Such a pattern is conserved between human and mouse. Gene overlap results in a small number of CFs (14 pairs) being found at the 3′-end of 3′UTRs. Additionally, the gene overlaps are evolutionarily conserved between human and mouse (Supplementary Table S3). However, these are a tiny fraction of the CFs found (28 out of 2905, see Table 1 above) near the 3′-end of the 3′UTRs. Other reasons are needed to explain the large number of CFs near the 3′-end.

### CFs are AT-rich and distinct from 5′UTR and CDS

The proportion of dinucleotides was examined across four sequence types: CDS, 5′UTR, 3′UTR, and CF. As CFs among the five core mammals are highly similar (>90% identity) by definition, only human sequences were examined here. The conclusion is expected to hold for other organisms.

**Figure 2.**
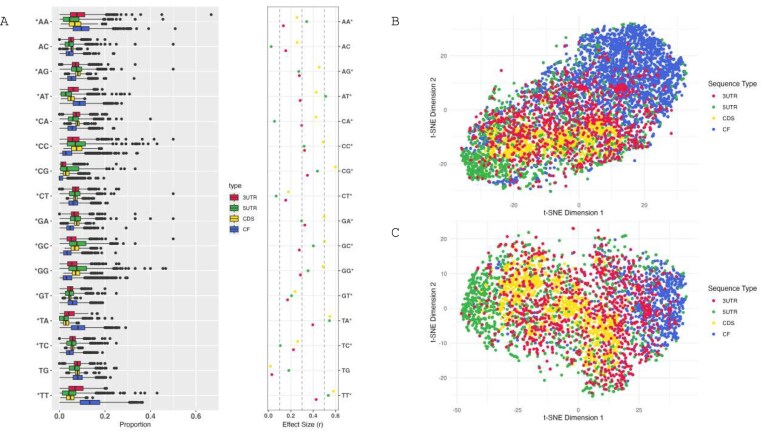
Nucleotide composition of CFs. (**A**) Dinucleotide composition of CFs when compared with 5′UTR, CDS, and 3′UTR in humans. The pairwise Wilcoxon Rank-Sum test was used to test differences of the medians of the four sequence types within a dinucleotide group. Statistically significant (adjusted *P*-value <.05) dinucleotides are marked with an asterisk (*). The effect sizes (the difference between CF and other sequence types) are shown on the right. (**B**) t-SNE plot for CF50 dinucleotides. CFs are clustered and overlap little with CDSs (yellow) and 5′UTRs (green). However, 3′UTRs (red) are scattered but distinct from 5′UTRs. (**C**) t-SNE plot for CF100 dinucleotides. CFs are more tightly clustered than CF50, suggesting that the sequence characteristics of longer CFs are distinct from shorter CFs. The t-SNE plot for CF200 is shown in Supplementary Figure S2.

The differences in proportions of dinucleotides were tested by the pairwise Wilcoxon Rank-Sum test. Dinucleotides marked by an asterisk in Fig. 2A indicate statistically significant. Noticeably, AT, TA, and TT dinucleotides have medians >75% quartile of other sequence types and have shown the largest effect sizes >0.5 (Fig. 2A). It is worth noting that, despite AA being the highest among sequence types in CFs, its median does not exceed the 75% quartile of the other three, and the corresponding effect sizes are <∼0.3, which are lower than those in AT, TA, and TT. These observations suggest that CFs are enriched by “T”s, and alternating T’s and A’s, instead of A-runs. Notably, based on the boxplot, no dinucleotide over- or underrepresentation is observed in the 3′UTR sequence type (red in Fig. 2A), even though they host the CFs.

Additionally, dinucleotides that exhibit an effect size >∼0.5 but are less abundant in CFs are CC, CG, GA, GC, and GG. This finding indicates these dinucleotides are depleted in CFs.

We further compared the nucleotide composition of CFs with other sequence types using the t-SNE plot. The plots for human CF50 and CF100 dinucleotides with 5′UTR, CDS, and 3′UTR are shown in Fig. 2B and C. The segregation between CF50’s CFs and CDSs is noticeable with minimal overlap, even more distinct for the CF100 group. The cluster of 5′UTRs is closer to the CDS, but distant from CFs, suggesting that the nucleotide composition of CFs is distinct from 5′UTRs and CDS. However, the cluster for 3′UTRs is closer to that of CFs, and yet they are distinguishable. The t-SNE plot for CF200 is provided in Supplementary Fig. S2.

### CFs are not low-complexity

Low-complexity and short repeats can lead to spurious sequence similarity. To eliminate such a possibility for CFs, we employ the idea of compressibility to assess whether CFs are composed of short repeating sequence units. Intuitively, if sequences are composed of simple repeats, they can be compressed to a greater degree than complex, non-repetitive sequences. We used the BPE compression method, which is widely used for tokenizing natural language. Here, we used the compression ratio attained by BPE as a measure of the complexity (or simplicity) of CFs. We benchmarked CF compression against real 5′UTR, CDS, and 3′UTR sequences. Additionally, we generated simulated sequences consisting of one to four different nucleotides: SIM1NT, SIM2NT, SIM3NT, and SIM4NT.

**Figure 3.**
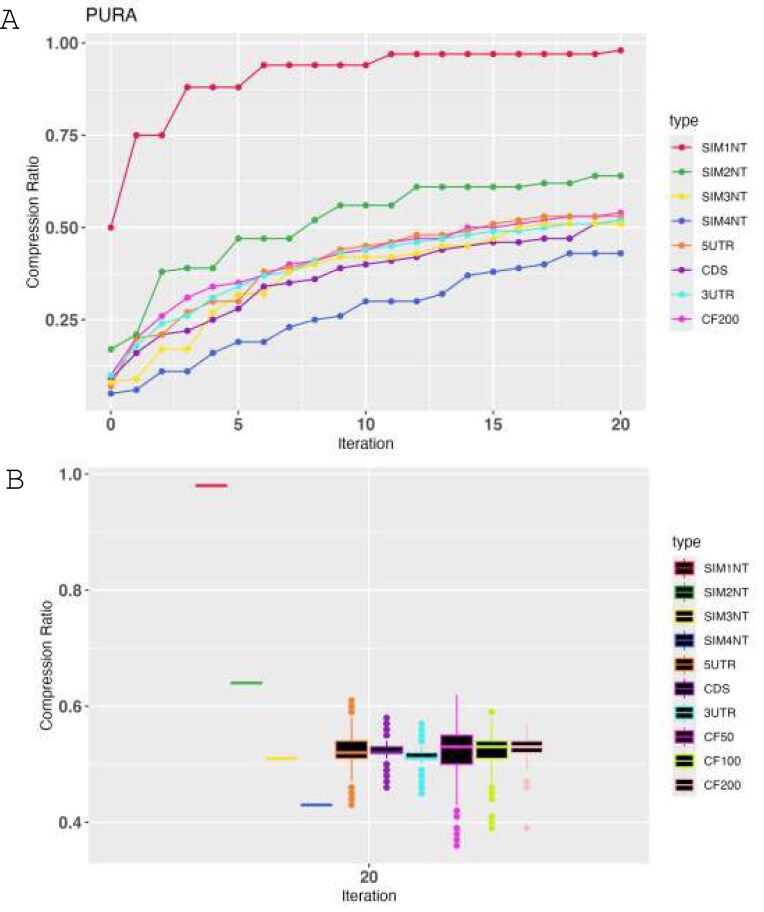
Low-complexity assessment by BPE. (**A**) An example of a particular gene, PURA. The degree of compression of the real sequences (5′UTR, CDS, 3′UTR, and CF of PURA) by iteration is similar to a simulated sequence consisting of three different nucleotides (SIM3NT). (**B**) Robust statistics of compression ratio for all sequences. The overall degree of compression of CFs from the three groups is similar to that of 5′UTR, CDS, and 3′UTR. Among the CFs, within-group variation reduces from the CF50 to the CF200 group, partly due to the sample size. Importantly, outliers are located below the lower whisker, indicating complex sequence patterns.

Figure 3A shows the trend in the compression ratio for various sequence types by iterations. Generally speaking, in a specific iteration, the compression ratio of a complex, non-repetitive sequence (e.g. SIM3NT and SIM4NT) is smaller than that of a sequence containing simple repeats (e.g. SIM1NT and SIM2NT). As shown, the compression ratio of the longest CF (from PURA) follows a similar trend to those of its CDS, 5′, and 3′UTRs, mirroring that of SIM3NT. It is evident that the CF from PURA is not derived from repeats.

Figure 3B compares all CFs with CDSs, 5′, and 3′UTRs at the 20th iteration or the end of compression, with simulated sequences as background. Differences in their median compression ratios were assessed using the Wilcoxon Rank-Sum test. The adjusted *P*-values and effect sizes are shown in Table 2. Note that the effect sizes were calculated using CF50 as the reference group. A large sample size of 5′UTR, 3′UTR, and CDS (see the “Materials and methods” section) can diminish *P*-values. Thus, supplementing *P*-values with effect sizes is a better approach for detecting real differences when sample sizes are large. According to common practice, an effect size of <0.1 indicates no meaningful difference. As shown in Table 2, the small effect sizes do not support meaningful differences between sequence types. While the largest difference is observed between 3′UTR and CF50, the effect size is only marginally above 0.1. Within CF groups, the median compression ratio remains similar, as supported by *P*-values and effect sizes. Within-group variation of CFs diminishes from CF50 to CF200 (Fig. 3), which is probably attributed to sample size and noise in shorter CFs. Nevertheless, outliers in CFs, regardless of group, are observed only at lower compression ratios, reflecting more complex sequence patterns across the three CF groups. To that end, BPE reviews that CFs are not dominated by simple repeats, and the sequence complexity of CFs is similar to 5′, 3′-UTRs, and CDS.

**Table 2.** Statistical analysis of compression ratios. *P*-values <.05 are bold

Adjusted *P*-value	5′UTR	CDS	3′UTR	CF50	CF100
CDS	0.400 88	-	-	-	-
3UTR	**<2e-16**	**<2e-16**	-	-	-
CF50	0.217 19	**0.018 45**	**<2e-16**	-	-
CF100	**0.011 54**	**0.000 26**	**<2e-16**	0.401	-
CF200	**0.037 85**	**0.0015**	**<2e-16**	0.401	0.554 04
Effect Size	5′UTR	CDS	3′UTR	CF100	CF200
CF50	0.0109	0.0176	0.112	0.0202	0.0251

To corroborate the compressibility analysis, we wanted to rule out the possibility that CFs are inundated with low-complexity repeats, which constitute 50% of the human genome [64]. RepeatMasker [40] was used to detect simple repeats and low-complexity sequences in CFs (based on the CF50 group), CDSs, and 3′UTRs. In all categories (SINE, LINE, LTR elements, DNA elements, unclassified, and interspersed repeats), 3′UTRs contain the highest percentage compared to CDS and CF (see Supplementary Table S4). More importantly, no LTR retrotransposons were found in CFs, eliminating the viral origin factor. CFs carry no satellites and have as few as 0.05% of low-complexity repeats, smaller than CDSs.

### CFs under the lens of transformer

At this point, we have learned that CFs are enriched with TT, TA, and AT dinucleotides. However, they are not saturated with low-complexity elements that are ubiquitous in the human genome. We are wondering which regulatory signals have been preserved in CFs over millions of years of evolution. Because their biological functions remain unknown and their lengths are much longer than typical sequence motifs, such as transcription factor binding sites, existing motif finders are not suitable for this task. Thus, we harnessed the DNA foundation language model GROVER [31] to unearth the sequence characteristics associated with the CFs. GROVER used the BPE mentioned above to tokenize the human genome into 609 tokens, including special tokens such as start, pad, etc., thereby easing model explainability analysis. Each token is a *k*-mer, where *k* ranges from 1 to 16. The shortest tokens of size 1 are individual nucleotides, and the longest tokens are a run of 16 A’s and Ts.

**Figure 4.**
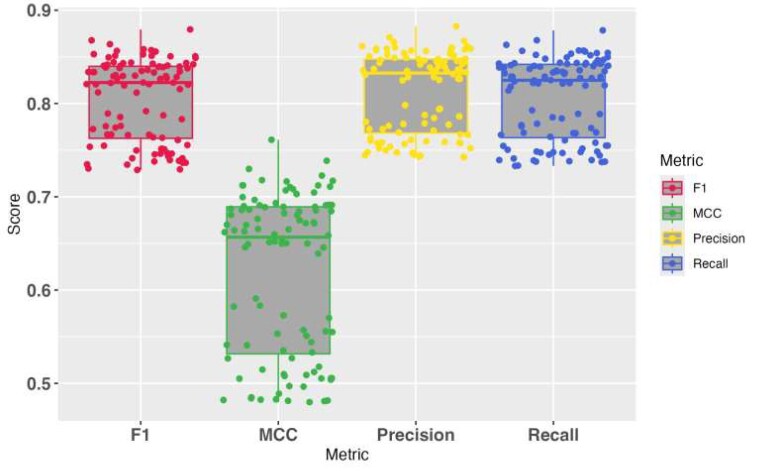
The accuracy of the GROVER-based CF model. The model was trained using five-fold cross-validation repeated 20 times. The median F1, MCC, Precision, and Recall are 0.82, 0.66, 0.83, and 0.82, respectively. Each dot represents a metric value from a test run. Five-fold cross-validation with 20 repeats generated 100 dots for each metric. The diamonds denote the medians of the holdout test metrics.

By using transfer learning, we built a binary classifier using the pre-trained GROVER model to distinguish CFs (*n* = 2989) from 3′UTRs that possess no CFs (*n* = 5203). The use of 3′UTRs as the negative samples is justified, as CFs originate from 3′UTRs. Factors that target CFs must distinguish between the two to enable specific interactions. To avoid issues with imbalanced data, the number of negative samples was kept equal to that of the positive samples via random sampling. 90% and 10% of samples were used for training plus validation, and holdout testing, respectively. Five-fold cross-validation was used during training. Evaluation statistics were obtained by training the model repeatedly 20 times. The model’s accuracy is assessed using F1, MCC, Precision, and Recall. For the five-fold cross-validation, the median F1, MCC, Precision, and Recall are 0.82, 0.66, 0.83, and 0.82, respectively (Fig. 4). For the holdout dataset, the median F1, MCC, Precision, and Recall are 0.74, 0.50, 0.75, and 0.75, respectively (diamonds in Fig. 4).

Based on the holdout results, the model has successfully distinguished the two sequence types and achieved F1, precision, and recall greater than 0.7. However, a considerable variation is observed in the MCC. The gap between F1 and MCC is attributed to two factors. First, F1 primarily focuses on predicting positives (i.e. CFs), while MCC integrates both positive and negative predictions (i.e. 3′UTRs with no CFs). The gap is attributed to the second factor, in which 3′UTRs are diverse. It is known that 3′UTRs are subjected to a weaker selective pressure than CDSs. Their diversity is also evident in the t-SNE plots in Fig. 2B and C. The heterogeneous nature of the negative samples, which are randomly sampled from a much larger 3′UTR space. This poses a challenge for the model in confidently predicting negative samples. Despite this, our objective is to identify signature sequences embedded in CFs, as F1, precision, and recall are all above 0.7, indicating that the model has captured sequence characteristics consistent with CFs. Lastly, the correlation between CF length and prediction scores was examined. It found no correlation (*r* = 0.02).

### Characteristics of CFs

The main purpose of building the classifier is to identify distinct sequence patterns captured by the model that differentiate CFs from non-CFs. The explainability method Integrated Gradients [45] was used to probe the model parameters and score the 604 GROVER tokens that contribute to its predictions, i.e. attribution. The average and median attributions of each token across the 10 repeated training runs were calculated and ranked (see Supplementary Table S5).

**Figure 5.**
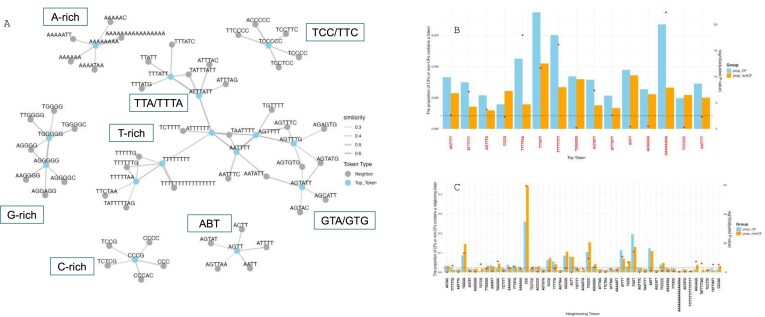
Explainability analysis. (**A**) A network of top 15 tokens (blue) identified by Integrated Gradients. The top tokens are connected to their five nearest neighbors (gray) based on GROVER’s embedding. The thickness of the edges represents cosine similarity. There are 64 tokens in total. Note that some top tokens are also neighbors of other top tokens, e.g. ATTTTTT and ATTTATT are top tokens and neighbors. (**B**) The proportion of CFs or non-CFs that contain a top token. The total number of CFs and non-CFs is 2989 and 5203, respectively. Top tokens are highlighted in red. The red dots represent the −log10 of adjusted *P*-values from proportion tests. The horizontal dashed line represents the adjusted 5% significance level after Bonferroni’s adjustment, i.e. −log10(0.05/64), which is equivalent to 3.1. (**C**) The proportion of CFs or non-CFs that contain a neighboring token. Same as panel (B) except that it is for the proportion of neighboring tokens in CFs and non-CFs.

The 15 tokens with the highest median attribution are colored in blue dots in Fig. 5A. To understand sequence variability, we also incorporated the five closest neighboring tokens of these 15 top tokens in GROVER’s embedding space, based on cosine similarity, and colored them in gray dots (Fig. 5A). In total, 64 tokens are considered. The thickness of edges represents cosine similarity (see Supplementary Table S6).

These tokens form into six clusters, indicating six distinct patterns are found in CFs. The biggest one is T-rich, in which T is the dominant nucleotide across all tokens, ranging from one-third to 100%. Ts are either punctuated or continuous. There is also a small cluster labeled ABT, where B means not A (i.e. G, C, or T) according to the IUPAC nomenclature. All tokens are shorter (4–5 bp) than other clusters, begin with A, and almost all contain two Ts, except for ATTTT. The A-rich and G-rich clusters constitute clearly homopolymeric A and G. Lastly, the C-rich and TCC/TTC clusters are enriched with a run of two to four Cs. Overall, these clusters provide critical information to understand the characteristics of CFs. Importantly, these 64 tokens, out of 604, provide specific patterns that can guide downstream analyses.

Moreover, we identified the proportions of positive and negative training sequences (including 100 bp padding) that contain the 64 tokens (Fig. 5B and C). Only sequences that are reliably predicted (prediction score ≥0.8) were selected and then tokenized. The top 15 tokens identified by Integrated Gradients above are highlighted in red in Fig. 5B. A proportion test was used to test the null hypothesis that tokens occur at the same level in CFs and non-CFs. The −log10 adjusted *P*-values are displayed for each token (red dots). Bonferroni’s adjustment was used to correct for multiple testing. The cutoff adjusted *P*-value (adjusted 5% significance level) for statistical significance is 0.05/64 or 3.1 after taking −log10 (horizontal dashed line in Fig. 5B and C). Tokens with a *P*-value above the cutoff among CFs and non-CFs indicate their differential prevalence, and they are tabulated in Table 3.

**Table 3.** Statistically significant tokens

CF		NonCF	
Token	*P*-value	Token	*P*-value
ATTTTTT	7.852089e-08	TGGGG	7.614483e-14
AGTTTG	1.907182e-04	TCTCG	2.896057e-06
TTTTTAA	1.126965e-18	TGGGGC	3.078462e-08
TTTATT	2.088561e-12	CCC	3.793659e-60
TTTTTTTT	7.045940e-17	TCCCC	4.174983e-10
AGTATT	4.548119e-08	AGGGGC	2.826885e-04
AAAAAAAA	4.731336e-23	TCCG	1.310195e-07
TTTTTTG	2.413110e-05	AGGAGG	3.934964e-08
TTTATG	1.606906e-04	TATTTTTAG	9.887097e-07
ATTTT	1.908592e-11	CCCAC	4.846523e-06
TTATT	7.763828e-11		
TATTTATT	5.093050e-06		

Consistent with the analysis above, T-, A-, and AT-rich tokens dominate CFs. On the other hand, tokens from the non-CFs are C- and G-rich.

### RNA secondary structures of CFs

Secondary structures across 3′UTRs are binding targets for RBPs, exerting vital regulation of stability, translation, and localization. We asked whether the secondary structures of CFs are distinct from those of background 3′UTR sequences. If a supervised classifier can distinguish them, revealing unique structural differences between the two. A 1D-CNN was built to answer this question. RNAfold [49] was used to predict the structures of CFs and non-CFs, using the same datasets that trained the GROVER model. Disappointingly, the median MCC ranged from 0.4 to 0.43 (Supplementary Fig. 3A and B), which was lower than that of the GROVER model.

Although the classifier results do not support that secondary structures alone are sufficient to offer a general characteristic of CFs, some CFs might still have prominent structures, i.e. both their sequences and structures are conserved. Based on the conserved RNA structures in the mouse from Seemann *et al*. [50], we mapped the conserved structures to mouse CFs from the CF50 group. Of the 2905 CFs from 1430 genes (Table 1), 201 CFs from 78 genes were found to entirely overlap with conserved structures (Supplementary Table S9). Notably, RAB1A is included, as it is a well-known example of a gene that exhibits conservation of sequence and structure in the 3′UTR [65].

We explored the shared tissue-specific expression, biological processes, and molecular functions of the human homologs of these 78 genes that harbor CFs and conserved structures. The R package Enrichr was used for the analysis [51]. We scanned all library names in Enrichr for the keyword “tissue,” yielding 10 libraries. But only these five produced positive enrichment results: ARCHS4_Tissues, Jensen_TISSUES, Descartes_Cell_Types_and_Tissue_2021, Tissue_Protein_Expression_from_Human_Proteome_Map, and GTEx_Tissue_Expression_Up. The proteome map is based on mass spectrometry, and the rest is from RNA-seq data. They are high-quality, comprehensive databases designed for tissue-specific analysis. Importantly, they are maintained by independent research groups, so aggregated results can be used to validate significant findings.

The top 10 enriched tissues, ranked by adjusted *P*-value ≤.05, are listed in Table 4. The complete list, with additional details, can be found in Supplementary Table S8. As shown, the majority of these genes are involved in neuronal tissues (OR > 3). When their enrichment of GO biological processes was examined (Table 5), four were neuronal processes, and three were related to transcription. In terms of molecular functions (Table 6), DNA/RNA binding and processing dominate. Results suggest that genes with CFs and conserved structures are associated with the transcriptional regulation of neuronal processes.

**Table 4.** Top 10 enriched tissues from genes with CFs and conserved structures

Term	Adj. *P*-value	Enriched genes	Genes	OR	95% CI
Temporal lobe	3.29E-13	63	8425	8.68	3.15–9.74
Occipital lobe	4.37E-12	66	10 045	9.25	2.80–9.61
Parietal lobe	9.48E-11	64	9921	7.33	2.48–7.89
Corpus callosum	2.41E-10	61	9077	6.25	2.41–7.08
Brain	3.09E-08	69	13 237	8.53	1.81–7.27
Frontal lobe	3.09E-08	65	11 591	6.09	1.88–6.20
Hypothalamus	3.17E-07	67	12 937	6.22	1.63–5.85
Cerebral peduncle	3.17E-07	40	4907	3.92	2.01–4.90
Eye	9.89E-07	42	5536	3.68	1.89–4.61
Embryonic brain	1.08E-06	39	4936	3.69	1.90–4.62

**Table 5.** The top 10 GO process enrichment of genes with CFs and conserved structures sorted by adjusted *P*-value

Term	Adj. *P*-value	Enriched genes	Genes	OR	95% CI
Neuron development (GO:0048666)	1.64E-03	7	159	12.48	5.65–27.58
Brain development (GO:0007420)	1.65E-03	7	170	11.63	5.27–25.67
Generation of neurons (GO:0048699)	2.02E-03	7	192	10.24	4.65–22.55
Negative regulation of cell cycle phase transition (GO:1901988)	2.02E-03	3	12	86.16	22.87–324.53
Cardiac muscle cell action potential (GO:0086001)	2.02E-03	4	37	31.71	10.96–91.77
Negative regulation of stem cell differentiation (GO:2000737)	2.14E-03	3	13	77.54	20.92–287.37
Regulation of transcription by RNA polymerase II (GO:0006357)	2.41E-03	22	2250	3.03	1.84–4.97
Neuron differentiation (GO:0030182)	5.52E-03	6	181	9.15	3.93–21.33
Positive regulation of DNA-templated transcription (GO:0045893)	5.52E-03	15	1274	3.43	1.95–6.04
Regulation of gene expression (GO:0010468)	5.52E-03	14	1127	3.59	2.01–6.43

**Table 6.** Enriched GO molecular functions

Term	Adj. *P*-value	Enriched genes	Genes	OR	95% CI
poly(A) binding (GO:0008143)	7.60E-03	3	24	36.90	10.78–126.35
Supercoiled DNA binding (GO:0097100)	7.60E-03	2	5	170.11	28.03–1032.50
Poly-purine tract binding (GO:0070717)	7.60E-03	3	30	28.69	8.52–96.62
miRNA binding (GO:0035198)	7.60E-03	3	30	28.69	8.52–96.62
Regulatory RNA binding (GO:0061980)	1.78E-02	3	43	19.36	5.86–63.94
mRNA binding (GO:0003729)	2.95E-02	6	310	5.23	2.26–12.13
DNA binding, bending (GO:0008301)	3.97E-02	2	17	34.00	7.64–151.24
Ankyrin binding (GO:0030506)	3.97E-02	2	18	31.87	7.20–141.02
Microtubule plus-end binding (GO:0051010)	3.97E-02	2	19	30.00	6.81–132.09
Voltage-gated monoatomic cation channel activity (GO:0022843)	4.47E-02	3	75	10.74	3.31–34.83

### CFs intolerant of variations in humans

Although the evolutionary approach we used, based on multiple sequence alignment, supports significant conservation across diverse species, it remains unclear whether CFs are intolerant of genetic variation due to vital biological constraints. Specifically, published genomes represent individual organisms; cross-species conservation, say >90% identity, does not guarantee within-species intolerance of variation, but within-species ultra-conservation foreshadows biological importance. To address this concern, we employed two independent metrics to assess the extent of variation constraint or depletion of variations in human CFs. The two metrics are JARVIS [66] and DR [46]. Both are based on large numbers of whole-genome sequences from healthy cohorts (62 784 for JARVIS and 150 119 for DR). The JARVIS score ranges from 0 to 1. 0 is perfectly tolerant of variation and 1 is totally intolerant of variation. To identify intolerant regions, the recommended JARVIS threshold is 0.9998, corresponding to the 99th percentile. DR also ranges between 0 and 1. However, 0 indicates the greatest depletion of variation and 1 indicates the least depletion of variation. For DR, the recommended threshold for detecting intolerant regions is ≤0.1.

**Figure 6.**
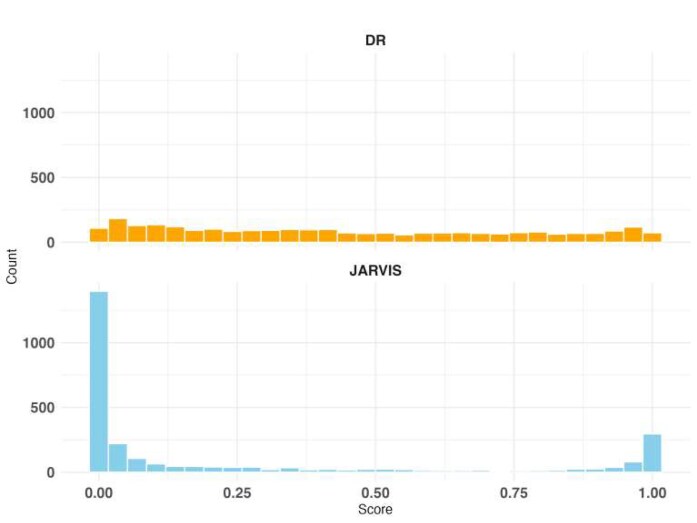
The median UK Biobank DR and JARVIS scores of CFs from the CF50 group (*n* = 2989). For the DR metric, scores ≤0.1 indicate depleted variation, i.e. the scores on the left of the orange plot. JARVIS scores ≥0.9998 indicate intolerant variation, corresponding to the scores on the right of the blue plot.

We applied these two independently established metrics to screen for intolerant CFs from the CF50 groups (*n* = 2989). JARVIS and DR scores were downloaded from UCSC GB for human hg38. The median score is determined for each CF, and their distribution is as shown in Fig. 6.

Next, we identify intolerant CFs with a JARVIS score of at least 0.9998 and a DR of no more than 0.1. As a result, 36 CFs from 25 genes were identified (Supplementary Table S9). We will refer to these as intolerant CFs and intolerant genes. Additionally, three intolerant genes (NRARP, SHANK3, and SOX21) also carry conserved RNA structures discussed above.

### Functional analysis of intolerant genes

Intriguingly, tissue enrichment analysis of these 25 intolerant genes revealed that all 31 tissues or samples are strongly associated (OR > 3) with the central nervous system (CNS), and no CIs have included 1.0 (no association) (Supplementary Table S9). Twenty-three of 25 intolerant genes are involved in the enriched tissues, except for H2AX and JunB. The top 10 enriched tissues ranked by adjusted *P*-value are highlighted in Table 7. Notably, 15 of 25 (or 60%) intolerance genes are enriched in the spinal cord, brain, and motor neurons, suggesting probable roles for intolerant CFs in neurons. When tissue expression of the 25 genes was examined from GTEx, all but four genes (CBX4, HOXA3, JUNB, and NRARP) exhibited elevated expression (median expression ≥90th percentile) in brain tissue samples.

**Table 7.** The top 10 enriched tissues of intolerant genes sorted by adjusted *P*-value

Term	Adj. *P*-value	Enriched genes	Genes	OR	95% CI
Spinal cord	1.09E-08	16	2316	13.25	5.85–30.03
Spinal cord (bulk)	1.09E-08	16	2316	13.25	5.85–30.03
Brain (bulk)	1.09E-08	16	2316	13.25	5.85–30.03
Motor neuron	6.34E-08	15	2316	11.18	5.02–24.91
Superior frontal gyrus	9.74E-07	14	2316	9.48	4.30–20.91
Oligodendrocyte	1.33E-04	11	2316	5.84	2.65–12.89
Fetal brain	1.40E-04	11	2316	5.84	2.65–12.89
Cerebral cortex	1.51E-03	10	2316	4.96	2.22–11.04
Cerebellum	1.77E-02	8	2316	3.49	1.51–8.11
Inhibitory interneurons in cerebellum	1.10E-02	2	33	54.47	12.31–241.00

Next, we performed a GO process enrichment analysis using the GO_Biological_Process_2025 database. Similarly, GO processes with an adjusted *P*-value ≤.05 are selected for analysis. Brain development, brain morphogenesis, camera-type eye development, neuron differentiation, and generation of neurons are the enriched processes (Supplementary Table S9). Out of the 15 genes involved in the process enrichment, only CBX4 and HOXA3 do not exhibit elevated expression in brain-related tissues in GTEx. This means that even for non-CNS-specific GO processes (regulation of DNA-templated transcription, branching morphogenesis of an epithelial tube, regulation of transcription by RNA polymerase II, regulation of cyclin-dependent protein serine/threonine kinase activity, stem cell differentiation, and positive regulation of kinase activity), the contributing genes are highly active in brain-related tissues. Because kinase activities are vital regulators of cell growth, metabolism, signaling, and division, they may be coupled to neuronal development.

Lastly, we investigated molecular function enrichment using the GO_Molecular_Function_2025 database. The results show that the enriched molecular functions are grouped into two categories: double-stranded DNA binding and kinase binding. These results align with the GO process enrichment. Nine of the 25 intolerant genes have contributed to the enrichment process, and all have exhibited expression above the 90th percentile in brain-related tissues. Such findings may suggest that these nine genes are involved in double-stranded DNA binding and kinase binding in neuronal tissues. The complete list of tissue, GO process, and GO molecular function enrichment results is provided in Supplementary Table S10.

### CFs of intolerant genes are expressed in brain and nerve tissues

As the enrichment analyses above were performed at the gene level, we wanted to confirm that CFs of the intolerant genes are expressed in the brain and nerve tissues. We manually confirmed that CFs are located in the last (3′-most) exon in the GTEx Portal. A custom R program was developed to retrieve the median expression levels of the last exons of the 25 intolerant genes across 54 non-disease tissues. Because the CFs are situated within the terminal exon, the expression level of this exon serves as a reliable proxy for the expression of the CFs themselves.

We shortlisted the top 10 tissues with the highest median expression (Supplementary Table S12), followed by tallying the number of brain or nerve tissues. As shown in Table 8, the majority of the intolerant genes (22/25) showed a strong tissue bias; notably, for 12 of these genes, at least 9 of their top 10 highest-expressing tissues were brain or nerve-related. The only exceptions were HOXA3, JUNB, and NRARP.

**Table 8.** Number of brain or nerve tissues in the top 10 highest-expressing non-disease tissues in GTEx for intolerant genes

Gene	Brain/nerve tissue	Gene	Brain/nerve tissue
AGAP2	10	MAPRE3	9
CAMK2N2	9	NRARP	0
CBX4	4	PAX2	5
CDK5R2	9	POU3F1	7
DLGAP3	10	SHANK3	6
FJX1	8	SNN	10
FZD9	7	SOX12	2
H2AFX	6	SOX21	7
HOXA3	0	TLX3	3
IRF2BP1	4	TMEM145	9
JUNB	0	VGF	9
LBX1	7	ZNF385A	7
LRFN1	9		

In summary, the enrichment analyses provide compelling evidence for a connection between genes harboring intolerant CFs and molecular processes involving the CNS. Potential processes encompass transcription regulation and kinase activity. GTEx results support the expression of CFs in neuronal or nerve-related tissues. Next, we will investigate factors that may exert functions upon binding to intolerant CFs.

### RNA-binding proteins and miRNAs bind to intolerant CFs

We are curious whether intolerant CFs are targets of RBPs and miRNAs, given that these are known to target 3′UTRs. We incorporated RNA-binding results from eCLIP experiments downloaded from the ENCODE Project [47], which included 250 RBPs tested on the K562 (*n* = 145) and HepG2 (*n* = 105) cell lines. Custom Python programs and bedtools [48] were used to map RBP binding sites and the 36 intolerant CFs to the human genome (hg38). In addition to RBPs, miRNAs are key regulators of gene expression by destabilizing mRNAs and repressing protein translation. These functions are exerted through binding to 3′UTRs. Hence, human predicted targets of conserved miRNA families from TargetScan [59] were mapped to the human hg38 genome. As a result, 25 out of 36 intolerant CFs are found to bind to miRNAs or RBPs or both (Table 9). Some genes appear more than once in the table because they harbor multiple intolerant CFs. More details, including CFs’ absence of RBPs and miRNA bindings, can be found in Supplementary Table S11. To determine the biological function of these interactions, we used GeneAgent [53], an LLM-based gene analysis tool, to elucidate interactions between the gene harboring the intolerant CF and miRNAs or RBPs. The primary reason we used GeneAgent rather than conventional GSA tools, such as Enrichr, is that they lack the novel relationship between intolerant CFs, miRNAs, and RBPs. Results are summarized in Table 9.

**Table 9.** Predicted interactions of intolerant genes, RBPs, and miRNAs by GeneAgent, an LLM-based GSA tool

Gene	Factor	Remark
DLGAP3	miR-331-3p	The evidence supports the role of DLGAP3 in modulating chemical synaptic transmission, but no direct interaction or combined regulatory mechanism between DLGAP3 and miR-331-3p is substantiated
	miR-101-3p.1, miR-101-3p.2, miR-144-3p, miR-27-3p, miR-128-3p	Unknown
PAX2	miR-133a-3p.1	miR-133a-3p.1 (MIR133A1) is a microRNA involved in post-transcriptional regulation of gene expression, modulating mRNA stability and translation. While its general regulatory role is established, there is no direct evidence linking miR-133a-3p.1 to optic nerve morphogenesis or PAX2 activity
H2AX	SUB1, SLBP, and PABPC4	H2AX contributes to chromatin integrity and DNA repair processes. Together, these proteins play complementary roles in histone mRNA metabolism and regulation, with SLBP being the central player in histone mRNA processing and the other genes contributing indirectly through chromatin maintenance, transcriptional activity, and mRNA stability
	miR-24-3p, miR-328-3p	The interaction between H2AX and miR-24-3p underscores a coordinated system in which histone modification and microRNA-mediated regulation converge to control DNA repair. This system is critical for the biological pathway associated with the NIPBL role in DNA damage in Cornelia de Lange syndrome
ZNF385A	DDX6, DGCR8, ZNF622	Together, DDX6 and DGCR8 play direct roles in post-transcriptional regulation, while ZNF385A and ZNF622 may contribute indirectly or through less-defined mechanisms
AGAP2	miR-302-3p/372-3p/373-3p/520-3p, miR-136-5p	Unknown
SOX21	miR-19-3p, miR-130-3p/301-3p/454-3p	Unknown
CBX4	DDX3X, HNRNPA1, PUM2, EFTUD2, DDX59, DHX30	The collective activity of these proteins demonstrates a coordinated system for RNA regulation and processing, particularly in splicing, transport, and translation. Their interactions ensure the fidelity of RNA maturation and gene expression, which are essential for cellular function.
JUNB	DROSHA, RBM15, DDX55	Together, these proteins form a system that integrates transcriptional regulation (JUNB), miRNA biogenesis (DROSHA), RNA modification (RBM15), and RNA structural remodeling (DDX55). This coordination ensures precise control of RNA processing and regulation, which is vital for cellular function and adaptation
LRFN1	miR-124-3p.1, miR-187-3p, miR-148-3p/152-3p, miR-124-3p.2/506-3p	Unknown
TMEM145	HNRNPK, DROSH	The interaction of DROSHA and HNRNPK suggests a coordinated system for regulating RNA metabolism, particularly miRNA biogenesis and RNA stability. DROSHA provides the enzymatic activity for miRNA maturation, while HNRNPK modulates RNA processing and stability. TMEM145 does not appear to contribute to these processes
RF2BP1	miR-340-5p	Unknown
	IGF2BP2	No functional or interaction data are available for RF2BP1, and its role in this system cannot be determined. Therefore, the biological process is primarily driven by IGF2BP2’s established functions in mRNA binding and translation regulation
	SUGP2, NKRF, TROVE2, LSM11, TAF15, GTF2F1, MTPAP, FXR2, DDX51, XRN2, DDX6, FUS, RBFOX2, NIPBL, CSTF2T, TIA1, DROSHA,SF3B4,NOLC1	This system demonstrates a tightly interconnected network of proteins that collectively regulate RNA metabolism, ensuring precise control of gene expression and cellular function
MAPRE3	miR-17-5p/20-5p/93-5p/106-5p/519-3p, miR-130-3p/301-3p/454-3p	MAPRE3 is critical for the regulation of mitotic spindle astral microtubule ends However, no direct evidence supports their involvement in mitotic spindle assembly or cytoskeletal remodeling
SOX12	miR-423-5p, miR-296-5p, miR-486-5p, miR-30-5p, miR-411-3p	Unknown
	DDX3X, SND1, HLTF, SMNDC1, NKRF, TROVE2, LSM11, PTBP1, SRSF1, GRWD1, GTF2F1, WDR43, CSTF2, DDX52, U2AF2, XRN2, PABPC4, NCBP2, DDX6, FUS, UCHL5, PCBP1, PCBP2, DGCR8, ILF3, STAU2, CPSF6, CSTF2T, FASTKD2, AKAP1, AGGF1, TIA1, PPIG, TBRG4, FTO, SDAD1, NOLC1, RPS3, PRPF4, BCCIP	This system of interacting proteins performs a coordinated set of functions essential for RNA processing and regulation, ensuring proper gene expression and cellular function
SHANK3	DDX3X, KHSRP, SUGP2, LARP4, EIF3D, SND1, IGF2BP3, SMNDC1, ZNF800, SRSF9, EXOSC5, SSB, TAF15, PTBP1, SRSF1, GRWD1, SLTM, SAFB, WDR43, MATR3, QKI, CSTF2, SRSF7, XRN2, EIF3H, NCBP2, BUD13, FUS, UCHL5, PCBP2, YBX3, RBM15, FASTKD2, AGGF1, RBM22, SDAD1	These proteins ensure the fidelity and efficiency of RNA processing, from splicing to translation, and integrate these processes with cellular signaling and stress responses
	SRSF1, KHSRP,U CHL5, GRWD1, ILF3, SRSF9	The convergence of these proteins on RNA-related processes highlights their roles in splicing, RNA stability, and translation regulation. SRSF1, SRSF9, KHSRP, and ILF3 are central to these functions, while UCHL5 and GRWD1 provide indirect regulatory support. SHANK3’s role remains peripheral to RNA processing. Together, this system ensures precise control of gene expression at the RNA level
	NCBP2, SRSF1, PCBP1, PPIG, GTF2F1, ZC3H11A, SRSF7, SMNDC1, GRSF1, TROVE2, SRSF9	This integrated network ensures the fidelity of gene expression at the RNA level
	miR-543	Unknown
	DDX6, DDX55, ILF3, SFPQ, EIF3D, HNRNPK, TIAL1, SRSF7	In summary, this system of interacting proteins collectively regulates RNA processing, stability, transport, and translation initiation. The presence of helicases, RBPs, and translation factors indicates a coordinated effort to control gene expression at the post-transcriptional and translational levels
CAMK2N2	miR-665, miR-122-5p, miR-377-3p, miR-483-3p.2	The system primarily integrates CAMK2N2’s role in synaptic plasticity with the general stress-regulatory functions of miR-665 and miR-377-3p, contributing to neuronal signaling and cellular homeostasis
	miR-17-5p/20-5p/93-5p/106-5p/519-3p	Unknown
	TROVE2,GTF2F1,CSTF2T	Together, GTF2F1, and CSTF2T are directly involved in transcriptional regulation and RNA maturation, while CAMK2N2 and TROVE2 may contribute indirectly to these processes. The interplay between these proteins supports the production of functional mRNAs and maintains gene expression fidelity
TLX3	miR-369-3p	Unknown
HOXA3	miR-15-5p/16-5p/195-5p/424-5p/497-5p	Unknown
	FASTKD2, CSTF2, ZC3H11A	The interaction of CSTF2 and ZC3H11A highlights their direct involvement in RNA processing and export, ensuring efficient mRNA maturation and transport. HOXA3 and FASTKD2 may contribute indirectly to these processes through transcriptional regulation and mitochondrial functions, respectively
VGF	miR-423-5p, miR-670-3p	The gene set shows significant enrichment in the hypothalamus, particularly in neuronal cells and projections, supporting its association with hypothalamic neuronal function. However, the specific interactions and mechanisms underlying VGF, miR-423-5p, and miR-670-3p in this context are not well defined
	DDX3X	RNA metabolism, including transcriptional regulation, mRNA export, translation initiation, and stress granule formation
NRARP	FMR1, TBRG4, NONO, PUM2, TROVE2, PABPC4, UPF1	This system collectively regulates mRNA stability, translation, and quality control, with each protein contributing to distinct yet interconnected aspects of post-transcriptional regulation

## Discussion

In this study, we have identified ~3000 CFs (≥50 bp, ≥90% identity) in the 3′UTRs of ∼1500 genes across three to five diverse mammals. Out of which, 30% of them are at least 100 bp long. They are enriched with AT, TA, and TT with respect to other sequence types: 5′UTR, CDS, and 3′UTR. And they are not composed of low-complexity or simple repeating sequences. In fact, their complexity is comparable to that of CDSs and 5′-/3′UTRs, eliminating low complexity as the reason for conservation.

AZIN1 and OAZ2 are key regulators of polyamine homeostasis, a tightly controlled process whose dysregulation has been implicated in diverse pathological conditions. Notably, both genes harbor CFs that exhibit deep conservation across highly divergent mammalian lineages, suggesting the presence of evolutionarily constrained regulatory elements. eCLIP data indicate that these CFs are bound by RBPs involved in mRNA stability and translational control, supporting a role in post-transcriptional regulation. Together, these observations illustrate how evolutionary conservation can guide the discovery of functional regulatory elements and highlight a promising framework for elucidating the biological roles of CFs.

CFs are found with a higher concentration at the two ends of 3′UTRs. With scant evidence at this moment, we speculate that these CFs might help prevent translation readthrough and polyadenylation, as AU-rich patterns resemble stop codons (UAA, UGA, and UAG) and canonical poly(A) signals (AAUAAA and AUUAAA).

Using a transformer-based genomic foundational model, GROVER, we have successfully identified distinctive tokens that distinguish CFs from the 3′UTR background. Based on the DNA embedding of the GROVER model, we have determined that signatory tokens are generally AT-rich, whereas non-CFs are G- and C-rich (Table 3). This information is essential for 3′UTR researchers, as AU-rich elements (AREs) in 3′UTRs are targets of AU-rich binding proteins, e.g. TTP and HuR (ELAVL1), which are most studied. A study has shown that translation efficiency can be elevated by introducing AREs [27, 67]. The HuR family has been reviewed recently to be associated with neurological disorders [68].

In addition to evolutionary conservation, we complement the functional annotation quest by examining intolerance of variation in humans. Using two stringent, independent metrics, 36 CFs from 25 genes were identified as under intense negative selection pressure. These genes are strongly associated with the CNS, involved in neurodevelopment, and regulate RNA transcription. Additionally, 22 of the 25 genes exhibit elevated tissue-specific expression in brain or nerve-related tissues. Based on eCLIP RNA-binding data and miRNA targets, RBPs and miRNAs that bind to the intolerant CFs play roles in transcription regulation and RNA metabolism. While we hypothesize that these CFs primarily serve CNS-related functions, our current RBP mapping is necessarily based on non-CNS cell lines because RNA-binding data are available only for them. Despite these tissue-specific constraints, these mapping results represent the most comprehensive data currently available for assessing RBP-CF interactions. As RBP and miRNA targets are usually short (<10 bp), the length of CFs may suggest combinatorial interactions by these factors, enabling diverse regulatory mechanisms [20].

RBP and miRNA bindings are influenced by RNA secondary structure. We have explored the predicted secondary structure of CFs by mapping them onto folded full-length mRNAs or folding CFs with 100-bp padding at both ends using RNAfold [49]. The CF folding structures produced by either method were fed into a convolutional neural network-based binary classifier to identify distinctive folding in CFs but not in non-CFs. However, no consistent features were found (Supplementary Fig. S3). It suggests that structure is not a primary reason for maintaining sequence conservation in CFs. Indeed, RNA structural conservation does not require sequence conservation. While such an attempt remained inconclusive, the structural aspect of CFs remains a critical factor. Therefore, we mapped known conserved RNA structures to CFs and found that 78 CF genes contained conserved structures. Intriguingly, the enrichment analysis indicates that they are expressed in the CNS, perform neuronal processing and transcriptional regulation, and, importantly, are similar to those of the intolerant genes.

In conclusion, this study suggests that a hundred CFs that are intolerant of variation and/or that carry conserved structures play essential neurodevelopmental roles in 3′UTRs. Previous studies of UCEs primarily focus on their enhancer function. A recent computational study hypothesizes a role for them in homologous DNA pairing [69]. Although 100 is a small number compared with the almost 3000 CFs reported here, our work establishes a link between CFs and RNA processing, implicating a critical role in neurodevelopment mediated by RBPs and miRNAs. These insights pave the way for high-throughput functional screening, similar to established enhancer assays [23], to advance our understanding of the biological functions of UCEs.

## Supplementary Material

lqag086_Supplemental_Files

## Data Availability

CFs identified in this study are kept in FASTA format. Multiple alignments by MAFFT for orthologous gene clusters are kept in compressed format. Additionally, the bed files contain human CFs mapped to the hg38 human genome. These files are available at NAR online.
